# Dynamic Consent for Secondary Use of Health Data: Challenges and Opportunities Under European Law

**DOI:** 10.2196/93348

**Published:** 2026-06-16

**Authors:** Sudip Phuyal, Manila Bhandari, Ricardo Correia Bezerra, Rabindra Bista, João Carlos Ferreira

**Affiliations:** 1 Information Sciences, Technology and Architecture Research Center (ISTAR), Iscte – Instituto Universitário de Lisboa, Lisbon, Portugal; 2 BioGHP - Global Health Platform S.A., Porto, Portugal; 3 Department of Computer Science and Engineering, Kathmandu University, Dhulikhel, Nepal; 4 Faculty of Logistics, Molde University College, Britvegen 2Molde, 6410, Norway, 47 71214000; 5 Inov Inesc, Lisbon, Portugal

**Keywords:** blockchain, dynamic consent, secondary use of health data, General Data Protection Regulation, GDPR, European Health Data Space, EHDS, interoperability, digital identity

## Abstract

Secondary use of health data is essential for advancing medical research, innovation, and public health policy across Europe. Traditional static or broad consent models are increasingly inadequate in complex, multistakeholder digital ecosystems. Dynamic consent, which enables granular, interactive, and ongoing management of individual preferences, including revocation, has emerged as a patient-centered alternative. This integrative review examines the legal feasibility and practical challenges of implementing dynamic consent for secondary health data use under the General Data Protection Regulation (GDPR) and the European Health Data Space (EHDS) Regulation. Drawing on doctrinal legal analysis, European policy documents, national derogations, and technical standards including Health Level Seven Fast Healthcare Interoperability Resources, electronic Identification, Authentication and Trust Services 2.0, European Digital Identity Wallet, and distributed ledger approaches, the study synthesizes legal, governance, and informatics perspectives. Findings indicate that while the GDPR establishes parameters supportive of specific, informed, and revocable consent, significant barriers persist due to national fragmentation, divergent lawful bases for processing, and limited cross-border revocation mechanisms. The EHDS, with provisions phasing in from 2029, shifts governance toward institutional authorization via Health Data Access Bodies and secure processing environments, reducing reliance on individual consent for many large-scale uses. Technical prerequisites, machine-readable consent artifacts, high-assurance digital identity, and policy-based enforcement remain unevenly developed. Nevertheless, integration with data altruism mechanisms under the Data Governance Act and emerging interoperability tools offers promising pathways. A 3-stage operational architecture (consent administration, decision, and enforcement) is proposed to embed dynamic consent within the hybrid EHDS-GDPR framework. However, challenges including blockchain immutability conflicts with the right to erasure, revocation propagation across systems, implementation costs, consent fatigue, and digital divides must be addressed. Dynamic consent cannot serve as a universal solution but can meaningfully enhance transparency and trust when deployed contextually alongside institutional safeguards. Coordinated EU-level harmonization, standardization, and inclusive design will be essential for its successful operationalization.

## Introduction

The secondary use of health data has become a structural feature of contemporary health research, digital innovation, and evidence-driven health care planning [1]. Large-scale datasets from electronic health records, registries, personal health apps, and population systems are now used for artificial intelligence, clinical decision support, public health surveillance, and device validation. This cross-institutional reuse raises governance challenges for rights protection and responsible innovation [2]. An assessment by the European Commission found substantial heterogeneity in Member State rules governing the reuse of health data, including differences in lawful bases, consent requirements, and access procedures across the European Union. This fragmentation affects the feasibility of consistent, cross-border mechanisms for obtaining and revoking consent and highlights the difficulty of designing harmonized governance models for secondary data use [3].

Consent is one of the most visible and debated mechanisms for legitimizing the processing of health data, especially for secondary uses beyond direct patient care [4,5]. Traditional models of consent are often static or broad, offering little scope for changing contexts associated with emerging secondary uses. The dynamic consent approach has emerged as a patient-centered alternative that facilitates ongoing engagement, real-time management of preferences, and significant revocability [6-8]. However, the dynamic consent mechanism is of uncertain feasibility within Europe. While the General Data Protection Regulation (GDPR) sets out requirements for valid consent on specificity, informed decision-making, and the ability to withdraw at any time [9], its practical implementation differs significantly across Member States and processing contexts. The regulatory landscape is further evolving with the introduction of the European Health Data Space (EHDS) Regulation, which seeks to harmonize secondary data use across the European Union. The EHDS introduces secure processing environments (SPEs), Health Data Access Bodies (HDABs), and a unified permit-based access model [10]. From a technical standpoint, operationalizing dynamic consent requires interoperable infrastructures, high-assurance digital identity (electronic Identification, Authentication and Trust Services 2.0 [eIDAS 2.0], European Digital Identity [EUDI] Wallet), verifiable credentials, privacy-preserving computation, and tamper-evident audit mechanisms, but these components vary widely in maturity across Member States [11].

This paper critically evaluates how European laws and infrastructures shape the feasibility of the implementation of dynamic consent for the secondary use of health data. It synthesizes the review from legal, organizational, and informatics perspectives to identify challenges and opportunities in bringing dynamic consent into action. It concludes by proposing a multilayered framework to guide future development and alignment efforts that will strengthen patient autonomy, transparency, and trust within Europe’s evolving digital health ecosystem.

Methodologically, this article adopts a doctrinal and integrative review approach [12,13], combining legal analysis of EU instruments governing the secondary use of health data, most notably the GDPR, the EHDS Regulation, the Data Governance Act (DGA), the Data Act, eIDAS 2.0, and selected national measures with a structured, nonsystematic review of relevant policy documents and interdisciplinary scientific and technical literature. The literature review was conducted using targeted searches in academic databases (eg, PubMed, Scopus, and Google Scholar) and institutional repositories, focusing on publications related to dynamic consent, health data governance, interoperability standards, and digital identity frameworks. Key search terms included combinations of “dynamic consent,” “secondary use of health data,” “GDPR,” “EHDS,” “health data governance,” and “consent management systems.”

Sources were selected based on relevance, recency, and contribution to legal, technical, or organizational aspects of dynamic consent. Priority was given to recent peer-reviewed articles (particularly from 2019 onward), European Commission reports, and standards documentation. While not intended as a PRISMA (Preferred Reporting Items for Systematic Reviews and Meta-Analyses)-style systematic review, sources were selected based on their relevance to 3 interrelated questions: the legal viability of dynamic consent under EU data protection law, the effects of national derogations and the EHDS governance model on the role of consent, and the technical and organizational preconditions for operationalizing dynamic consent in cross-border health data ecosystems. The analysis followed a qualitative synthesis approach, integrating legal interpretation with technical and policy perspectives to identify key barriers, enablers, and implementation pathways. The article presents no empirical data; its contribution is normative and conceptual.

## Regulatory Background and European Legal Context

### Overview

The feasibility of dynamic consent for secondary use of health data in Europe is primarily shaped by the interaction between the GDPR, the emerging EHDS Regulation, and a set of related legal instruments and national derogations. Together, these norms define the conditions under which health data may be reused, the role and limits of individual consent, and the governance responsibilities of controllers, processors, and dedicated access bodies.

### GDPR and the Role of Consent in Secondary Health Data Use

The GDPR forms the general legal framework for personal data processing within the European Union. Health data are classified as a special category of personal data under Article 9(1), meaning that their processing is, in principle, prohibited unless one of the conditions listed in Article 9(2) applies. Explicit consent of the data subject is one such condition. Where processing is based on consent, the GDPR requires that it be freely given, specific, informed, and unambiguous, and that withdrawal be as easy as giving consent, without affecting the lawfulness of prior processing.

In principle, dynamic consent aligns closely with these requirements. By enabling ongoing, granular, and interactive management of consent preferences, dynamic consent promises to bring actual practice closer to the normative standards articulated by the GDPR. It allows individuals to refine their consent over time, to respond to new secondary-use requests as they emerge, and to exercise withdrawal in a manner that is more than merely theoretical.

At the same time, the GDPR also recognizes alternative lawful bases for processing health data in secondary-use contexts. Articles 9(2)(i) and 9(2)(j), read in conjunction with Article 6, allow processing where necessary for reasons of public interest in the area of public health or for scientific or historical research purposes, subject to appropriate safeguards, including pseudonymization and data minimization. In many Member States, these research and public-interest grounds are relied upon more frequently than consent in large-scale secondary-use infrastructures, thereby limiting the centrality of consent in practice.

### Conditions for Valid and Revocable Consent

The GDPR’s conditions for valid consent have important implications for digital consent mechanisms. First, consent must be specific and granular with respect to the purposes of processing. Blanket authorizations for “future research” without adequate specification are difficult to reconcile with these requirements. Second, consent must be informed and based on transparent information about the nature of processing, categories of recipients, and risks involved. Third, the regulation emphasizes that consent must be revocable at any time, and that withdrawal should be as easy as the act of giving consent.

From a legal perspective, these characteristics favor digital, interactive systems such as dynamic consent platforms, which can express user preferences as structured data, link them to specific purposes, and provide user-friendly tools for modification and withdrawal. However, the GDPR is largely silent on how revocation should operate in distributed or cross-border data environments. It does not specify how withdrawal should be propagated to downstream users, how it should be applied to derived or aggregated datasets, or how controllers should handle revocation once data have been incorporated into trained machine learning models or long-term research cohorts. This lack of operational guidance creates uncertainty and contributes to heterogeneous practices across Member States and sectors. To contextualize these challenges, different consent models used in Europe can be broadly distinguished based on their level of granularity, adaptability, and support for revocation. Traditional models such as static, broad, and tiered consent provide limited flexibility and weak mechanisms for ongoing user control, whereas dynamic consent enables continuous, granular, and interactive management of consent preferences over time. Accordingly, dynamic consent is presented as a more flexible and responsive model that better aligns with GDPR requirements for specificity, transparency, and ease of withdrawal. For this reason, the remainder of this article focuses specifically on dynamic consent as the most relevant and future-oriented model for secondary health data use in Europe.

### Fragmentation Introduced by National Derogations

Although the GDPR seeks to harmonize data protection law across the European Union, it explicitly allows Member States to introduce additional rules concerning the processing of special-category data, including health data, under Article 9(4). Many Member States have exercised this discretion, adopting national provisions that specify or constrain the use of consent and research exemptions in the health sector.

The result is a structurally fragmented legal landscape. In some jurisdictions, broad secondary-use permissions are available under detailed statutory frameworks; in others, strict conditions are imposed on research uses, including additional ethical approvals or sector-specific authorization procedures. Definitions of “explicit consent,” the scope of research exemptions, and the allocation of governance responsibilities between ethics committees, data protection authorities, and specialized access bodies vary across countries. For cross-border research projects, this means that a consent model considered valid and sufficient in one Member State may not be regarded as adequate in another.

For dynamic consent, national derogations pose a particular challenge. A digital platform that offers a unified interface for managing consent across multiple Member States must nonetheless comply with divergent national rules on when consent is required, which alternative legal bases are available, and how withdrawal must be handled. This greatly complicates efforts to design a single, harmonized dynamic consent model that would function uniformly across the European Economic Area [3].

### The EHDS and the Governance Shift for Secondary Use

The EHDS Regulation introduces a sector-specific framework for the secondary use of electronic health data at the EU level. It establishes HDABs with a mandate to receive applications for access to health datasets, to issue data permits under harmonized conditions, and to ensure that processing takes place within SPEs. The regulation defines a list of permissible secondary-use purposes, including scientific research, innovation, public health, policy-making, and regulatory activities, and sets out safeguards such as data minimization, prohibition of attempts at reidentification, and restrictions on data export.

Within this architecture, the locus of governance for many secondary-use scenarios shifts from the individual level to institutional authorization. While the GDPR remains applicable, and consent continues to be one possible lawful basis, the EHDS effectively foregrounds public interest and research grounds, mediated through HDAB permits and SPEs, as the principal mechanisms for legitimizing large-scale secondary-use processing. In such settings, data subjects do not necessarily provide consent for each specific secondary-use project; instead, they rely on institutional safeguards and regulatory oversight.

This governance shift does not render consent irrelevant, but it does reconfigure its role. Consent becomes context-specific rather than the default authorization mechanism for all secondary uses. It remains more salient in settings where individuals actively contribute data (eg, via patient-held records or personal health apps) or where national law insists on consent for categories of secondary use than in the institutionalized infrastructures envisaged by the EHDS.

Importantly, this governance model should be understood within the EHDS implementation timeline rather than as an immediately operational infrastructure. Although Regulation (EU) 2025/327 formally entered into force in 2025, many of its operational provisions for secondary use, including the establishment of HDABs, interoperability obligations, and SPE requirements, will be implemented progressively, with major obligations applying from 2029 onward and some cross-border capabilities expected to mature later. During this transitional period, Member States are likely to continue relying on heterogeneous national governance arrangements, legacy health data platforms, and existing research authorization mechanisms. This transitional reality has important implications for dynamic consent: in the short to medium term, implementation will need to coexist with fragmented national infrastructures and uneven technical readiness, while the architecture proposed in this article should be understood as a phased model that aligns with the gradual institutional maturation of the EHDS rather than an immediately deployable EU-wide framework.

### Supporting Frameworks: Digital Identity, Data Governance, and Ethics

In addition to the GDPR and EHDS, several other European instruments shape the operational environment within which dynamic consent would have to function. The DGA creates a framework for data intermediaries and data altruism organizations, which rely on clear and revocable consent for the reuse of data, including health data, for the benefit of society. The Data Act, in turn, introduces horizontal rules on access to and use of data, including user rights to share and permit third-party access to data generated by connected products and related services.

The revised eIDAS 2.0 framework and the planned EUDI Wallet provide an emerging legal basis for high-assurance digital identity and verifiable credentials. These instruments are directly relevant to dynamic consent, as they can support reliable authentication of data subjects and cryptographic binding of consent artifacts to specific persons. They also establish the conditions under which identity credentials must be recognized across borders within the European Union.

Ethics committees, national research governance bodies, and sector-specific regulations (eg, clinical trials or public health surveillance) further influence when consent is required, how it is documented, and which safeguards must accompany consent-based or consent-free processing. Even where the legal basis is not consent, such bodies often insist on transparency and engagement measures that resemble certain aspects of dynamic consent, such as providing accessible information on data reuse and offering opportunities to express preferences or objections.

In this respect, the DGA is particularly relevant not merely as background legislation, but as a governance instrument that operationalizes data intermediation and data altruism through structured permission, transparency, and organizational accountability. For dynamic consent, this is significant because it provides a legal and institutional context in which individuals may voluntarily make data available for socially beneficial reuse while retaining a meaningful role in permission-setting and trust formation. Accordingly, the DGA can be understood as complementing the GDPR’s rights-based consent model and the EHDS’s permit-based access model, especially in settings involving patient-mediated data sharing and data altruism organizations.

### Implications for Cross-Border Secondary Data Use

The combined effect of GDPR, national derogations, and the EHDS is a complex, hybrid governance landscape for cross-border secondary use of health data. On the one hand, the GDPR establishes a common set of principles and rights, including conditions for valid consent and the right to withdraw. On the other hand, Member States’ additional rules for health data processing, alongside the sector-specific authorization model introduced by the EHDS, create significant variation in how those principles are implemented.

For cross-border research consortia, registries, or data spaces, this hybrid landscape raises several questions. A consent decision given in one jurisdiction may not be sufficient in another, either because local law requires additional safeguards or because the lawful basis relied upon is not consent at all but a research or public-interest ground. Mechanisms for recognizing and enforcing withdrawal across borders are not standardized, and the institutional responsibilities for handling revocation signals are distributed across multiple actors, including controllers, processors, HDABs, and SPE operators.

In this context, dynamic consent faces both legal and practical obstacles. To function as a meaningful governance tool in cross-border settings, it would need to be compatible with divergent national rules, align with EHDS authorization procedures, and be supported by interoperable technical infrastructures capable of capturing, representing, and propagating consent decisions across jurisdictions and institutions.

### GDPR and EHDS: The Legal Hierarchy and the Role of Consent

The relationship between the GDPR and the EHDS can be conceptualized as one of *lex generalis* and *lex specialis*. The GDPR remains the general data protection framework applicable to all processing of personal data, including health data, in the European Union. It defines the core principles of data processing, the catalog of data subject rights, and the conditions under which consent is valid and revocable. In this capacity, it continues to govern both primary and secondary use of health data, regardless of whether processing occurs under national law or within the structures established by the EHDS.

The EHDS, by contrast, operates as *lex specialis* for specified categories of secondary use of electronic health data. It does not replace the GDPR but overlays it with sector-specific rules on governance, access, and safeguards. In practice, this means that, for many EHDS-governed secondary uses, the lawful basis for processing will be derived from public-interest and research grounds, as specified in Articles 6 and 9 GDPR and in national implementing legislation, rather than from consent. Consent remains legally available, but it is no longer the presumptive default in all secondary-use scenarios.

In light of this hierarchy, dynamic consent should be understood as a context-dependent mechanism whose deployment must be carefully aligned with both the GDPR and the EHDS. It is most promising in domains where individuals actively manage or contribute their own data, such as patient-controlled health records, personal health apps, citizen-generated data ecosystems, and data altruism initiatives, and where national law or institutional practice maintains a strong role for consent. In large-scale, EHDS-governed infrastructures, dynamic consent is more likely to operate as a complementary measure enhancing transparency and participation, rather than as the primary legal basis for processing. The following sections build on this understanding to examine how dynamic consent might realistically be integrated into Europe’s evolving health data governance regime.

## Critical Analysis: Legal Barriers and Enablers of Dynamic Consent

### Overview

Legal, governance, and technical factors are all interlinked in terms of how dynamic consent for secondary use of health data in Europe is laid down. Even though the GDPR lays down a sound rights-based framework, the practical role of consent is limited due to such factors as fragmented national interpretations of consent under the GDPR, reliance on alternative lawful bases to consent, and the governance model devised by the EHDS. Dynamic consent is also operationally hindered by factors such as the absence of effective revocation mechanisms, partial and inconsistent adoption of technical standards, and gaps in cross-border interoperability. The next section critically examines those constraints and enabling conditions to establish the basis for the implementation framework discussed in the “Implementing Dynamic Consent in Europe” section.

### GDPR: Alignment and Operational Ambiguities

The GDPR aligns conceptually with dynamic consent. Its requirements for specificity, informed decision-making, transparency, and ease of withdrawal correspond directly to the capabilities of digital, interactive consent systems. Dynamic consent can embody GDPR’s rights more powerfully than static models because it enables participants to manage preferences over time and receive updates on an ongoing basis about data use.

However, the main challenges arise from the significant operational ambiguity of GDPR. The regulation does not specify how revocation should propagate across distributed or federated systems, nor does it detail how withdrawal applies to data already processed, pseudonymized, or integrated into derived datasets. This lack of operational clarity contributes to inconsistent practices across sectors and Member States, which constrain the use of consent as a robust and enforceable legal basis in secondary-use research infrastructures.

The Commission’s findings further substantiate this ambiguity: although GDPR establishes a coherent rights-based framework, its practical application diverges widely across national legal systems [3]. Stakeholder feedback collected in the assessment describes irregular and sometimes burdensome procedures for authorizing secondary-use research, reinforcing that operationalizing dynamic consent requires navigating a complex and often inconsistent regulatory environment.

### Fragmentation Caused by National Legal Derogations

Fragmentation in national rules governing the secondary use of health data has been empirically documented in the European Commission–commissioned assessment of Member State rules on health data processing under the GDPR [3]. Based on national legal mapping, expert surveys, and stakeholder consultations across all EU Member States, the study demonstrates the existence of markedly divergent national regimes governing secondary use of health data, including differences in the role attributed to consent, the scope of research exemptions, and the structure of data access governance.

A major structural barrier arises from Article 9(4) and related national provisions that allow Member States to introduce additional safeguards or conditions for the processing of health data. This has resulted in a patchwork of national rules governing what constitutes explicit consent, when consent is required, and which alternative lawful bases may substitute for consent in research contexts.

Examples include:

1. Finland: broad secondary-use permissions introduced through the Secondary Use Act [14]
2. Germany: strict restrictions with respect to research use; further ethical approvals required [15]
3. France: heavy dependence on institutional frameworks for data access [16]
4. Norway (European Economic Area): project-level approvals rather than consent-driven governance [17]

This heterogeneity makes cross-border research more complicated under GDPR due to inconsistent expectations of consent and revocation. It also makes it nearly impossible to implement a harmonized, EU-wide dynamic consent model, since a consent decision assumed meaningful in one jurisdiction may not hold equivalent legal status in another. Fragmentation therefore constitutes one of the most significant barriers to scalable dynamic consent for secondary health data use.

### Practical Limits of Revocability

While revocability is a constituent characteristic of dynamic consent, its possible application is practically limited by scientific, technical, and ethical reasons.

Key challenges include the following:

1. Irreversible data uses, such as inclusion in aggregated datasets or trained machine learning models
2. Pseudonymization practices that make targeted retrieval or erasure of individual records difficult or impossible
3. Scientific integrity requirements where the removal of data retrospectively may undermine validity or reproducibility
4. Downstream data flows where the revocation signals do not propagate effectively across institutional or cross-border collaborations

These challenges reflect a continuing tension between the rights-based framing of GDPR and the actualities of contemporary data processing.

A further legal distinction is essential here: pseudonymized data remain personal data under the GDPR because they can still be linked to an identifiable individual through additional information held separately, whereas truly anonymized data fall outside the scope of EU data protection law once reidentification is no longer reasonably possible. This boundary has direct implications for dynamic consent. Where health data have only been pseudonymized, data subject rights, including withdrawal of consent where consent is the relevant legal basis, continue to apply. By contrast, once data have been rendered effectively anonymous in a legally meaningful sense, the GDPR no longer applies to that dataset, and the right to withdraw consent no longer operates in relation to its subsequent use as personal data. In practice, however, fully anonymizing health data is often difficult, especially in longitudinal, genomic, rare-disease, or highly linkable datasets. For this reason, many secondary-use environments remain governed by the law of personal data even where strong deidentification techniques are used.

### EHDS: Governance Shift and Implications for Consent

The EHDS Regulation introduces a harmonized framework for secondary health data use based on regulated institutional access. The EHDS aims to achieve transparency and consistent oversight through HDABs, SPEs, and standardized permit procedures.

While these infrastructures strengthen governance, they also reconfigure the role of consent. Consent becomes context-specific rather than the dominant authorization model, particularly for large-scale analytics and population-level research. Dynamic consent therefore remains relevant in scenarios where individuals directly contribute or control their own data, such as patient-held records, cross-border care interactions, and participatory research, but is less central to the EHDS’s institutionalized secondary-use framework.

Notably, the Commission report [3] also documents that several Member States already rely on centralized or semicentralized data access bodies for health data research, structures that closely resemble the governance model anticipated by the EHDS. This trend indicates that institutional authorization is becoming more prominent than individual consent for large-scale secondary-use scenarios, strengthening the argument that dynamic consent will operate most effectively in contexts where individuals directly manage or contribute data.

### Technical Feasibility Gaps

Dynamic consent presupposes a set of technical and organizational capabilities that, at present, are unevenly developed across Europe. At least 4 areas are particularly salient.

First, consent representation and interoperability. Although standards such as the Health Level Seven (HL7) Fast Healthcare Interoperability Resources (FHIR) Consent resource [18] provide a structured way to encode consent decisions, their adoption in national eHealth infrastructures remains partial and heterogeneous. In many health systems, consent is still captured in unstructured formats (PDF forms and free-text electronic health record entries) that cannot readily be processed by policy engines or propagated across institutional boundaries. Without widely implemented, machine-readable consent artifacts, the granular preference management that dynamic consent entails cannot be reliably automated.

Second, digital identity and authentication. Dynamic consent hinges on the ability to authenticate data subjects with sufficient assurance and to bind their decisions to cryptographically verifiable identifiers. The revised eIDAS 2.0 framework and the planned EUDI Wallet provide a legal and technical basis for this, enabling cross-border recognition of high-assurance identity credentials. However, health-sector integration of these tools is still at an early stage, and many Member States rely on fragmented identity solutions that are not interoperable. This undermines the possibility of a single, EU-wide mechanism through which individuals could manage consent for secondary use of health data.

Third, policy-based access control and enforcement. Dynamic consent requires that consent artifacts be translated into enforceable authorization rules at the level of data access. While policy engines and “policy-as-code” approaches, including attribute-based access control, Open Policy Agent, and eXtensible Access Control Markup Language–style frameworks, are increasingly used in other domains, they are not yet systematically deployed in health research infrastructures and SPEs. As a result, many data access decisions continue to be governed by static procedures and manual review, with limited capacity to reflect individual, dynamically changing consent preferences in real time.

Fourth, logging, auditability, and revocation propagation. To make revocation effective, systems must be capable of logging all access requests, linking them to specific consent artifacts, and propagating withdrawal signals across distributed data environments. SPEs envisaged by the EHDS are expected to provide detailed logging and monitoring capabilities, but these are not yet uniformly available. Approaches based on Distributed Ledger Technology have been proposed to provide tamper-evident audit trails and consent provenance [19], yet they remain largely experimental.

Taken together, these gaps illustrate that the technical prerequisites for dynamic consent are only partially in place. While the individual building blocks FHIR-based consent artifacts, EU-level digital identity, policy engines, and tamper-evident auditing are emerging, their integration into a coherent, cross-border infrastructure for health data governance remains incomplete. This technological immaturity is a key factor limiting the practical feasibility of dynamic consent at scale.

### Synthesis

The analysis in this section reveals a dual tension at the heart of dynamic consent for secondary use of health data in Europe. Normatively, the GDPR is highly compatible with dynamic consent: it emphasizes specificity, transparency, and the ability to withdraw, and it treats consent as one possible lawful basis for processing special-category data. In addition, emerging technologies suggest that granular, machine-readable consent could in principle be implemented through interoperable artifacts, high-assurance digital identity, and policy-based enforcement mechanisms.

Practically, however, the scalability of dynamic consent is constrained on 3 fronts. National derogations under Article 9(4) GDPR have produced a structurally fragmented legal environment, in which the role of consent and the scope of research exemptions vary significantly between Member States. The EHDS, acting as lex specialis, introduces a governance architecture that privileges institutional authorization and SPEs over individual consent for many forms of secondary use. And key technical components, interoperable consent standards, identity integration, policy engines, and revocation-aware audit infrastructures are not yet mature or harmonized across the Union.

Dynamic consent, therefore, cannot be treated as a universal solution for secondary health data use. Its deployment must be targeted at contexts where it is both legally viable and operationally meaningful, and it must be embedded within the broader institutional architecture created by the EHDS. The next section develops an implementation framework that is expressly designed to reflect these constraints, focusing on how dynamic consent could function as a complementary mechanism within the emergent EU health data regime.

Table 1 synthesizes the main legal, governance, and technical barriers identified in the preceding critical analysis, together with potential enablers discussed in the literature.

**Table 1.** Summary of barriers and potential enablers affecting the feasibility of dynamic consent in Europe.

Category	Key barriers	Implications	Potential enablers
Legal (GDPR^a^)	Ambiguity around revocation propagation; unclear handling of derived datasets	Inconsistent withdrawal practices across institutions	European Data Protection Board guidance; harmonized EU^b^ interpretations
National derogations	Divergent national rules on explicit consent and research exemptions	Fragmented cross-border governance	EHDS^c^ harmonization; coordinated legislative alignment
Governance (EHDS)	Shift toward institutional authorization over consent	Consent becomes context-dependent	Consent-aware policy engines within SPEs^d^
Revocability	Irreversible use in models and datasets; pseudonymization; difficulty of achieving legally robust anonymization	Limited effectiveness of retrospective withdrawal and uncertainty regarding when GDPR rights cease to apply	Prospective revocation; privacy-preserving computation; clearer legal guidance on anonymization thresholds
Identity layer	Fragmented digital identity systems	Weak attribution of consent decisions	EUDI^e^ Wallet; verifiable credentials; decentralized identifiers
Interoperability	Low adoption of HL7^f^ FHIR^g^ Consent	Hard to encode and enforce granular consent	EU-wide FHIR Consent profiles
Enforcement	Limited deployment of policy engines	No real-time enforcement across systems	Standardized consent-aware authorization (eg, OPA^h^ and XACML^i^)
Auditability	Inconsistent logs outside SPEs	Reduced transparency and accountability	Distributed ledger auditing; SPE logs

a GDPR: General Data Protection Regulation.

b EU: European Union.

c EHDS: European Health Data Space.

d SPE: secure processing environment.

e EUDI: European Digital Identity.

f HL7: Health Level Seven.

g FHIR: Fast Healthcare Interoperability Resources.

h OPA: Open Policy Agent.

i XACML: eXtensible Access Control Markup Language.

## Implementing Dynamic Consent in Europe

### Overview

Implementing dynamic consent in the European setting will need alignment of legal norms, digital identity infrastructures, standards for interoperability, and governance structures. While there are many hindrances—which have been discussed in the previous section—several developments within the GDPR, EHDS, and ongoing digital identity frameworks do present real opportunities for embedding dynamic consent in European secondary-use ecosystems. This section elucidates such opportunities and proposes a structured multilayer framework that can be adapted for implementation.

### Opportunities Within the Emerging European Health Data Infrastructure

Although the preceding analysis highlights substantial constraints, it also indicates that the evolving EU health data infrastructure contains several levers that could support dynamic consent in appropriate contexts. The GDPR provides a rights-based baseline in which consent, when used, must be specific, informed, and revocable, and in which individuals enjoy ancillary rights of access, rectification, restriction, and objection. This framework ensures that any dynamic consent mechanism is anchored in robust individual rights protections rather than merely contractual arrangements.

The EHDS, for its part, introduces harmonized institutional structures, HDABs, SPEs, and standardized permit procedures that can be configured to incorporate consent-aware authorization rules where consent remains a relevant lawful basis. Although the EHDS does not mandate dynamic consent, its requirements regarding logging, data minimization, nonexport, and secure computation create an environment in which individual consent preferences, when captured in interoperable form, could be enforced with a higher degree of reliability than is currently possible in many decentralized settings.

Parallel initiatives in digital identity and technical standardization also open up new implementation pathways. The EUDI Wallet promises cross-border identity assurance and the issuance of verifiable credentials that could be used to authenticate consent decisions and bind them to specific data subjects. Likewise, continued work on HL7 FHIR, including efforts to profile FHIR Consent for EU use cases, offers a potential lingua franca for encoding and exchanging consent artifacts across health information systems. Finally, increasing interest in privacy-enhancing technologies and policy-as-code approaches in research infrastructures lays the groundwork for more automated and transparent enforcement of consent conditions.

The DGA also adds an important organizational layer to this emerging infrastructure. In particular, its provisions on recognized data altruism organizations and trusted data intermediation services create institutional arrangements through which individuals may authorize socially valuable reuse of data under more transparent governance conditions. In practice, this means that dynamic consent could be embedded not only in direct controller–data subject relationships, but also in intermediary settings where consent preferences, participation choices, and reuse conditions are mediated through recognized governance actors. This strengthens the relevance of dynamic consent in participant-driven and altruism-based data-sharing models, even where EHDS-style institutional authorization governs other forms of secondary use.

These developments suggest that dynamic consent should not be dismissed as impracticable. Rather, it should be reconceptualized as a mechanism that can be selectively deployed within and supported by the institutional and technical architecture established by the EHDS and related initiatives. The 3-stage framework set out in the following subsections is designed precisely with this hybrid governance model in mind.

### Operational Architecture for Dynamic Consent

**Figure 1.**
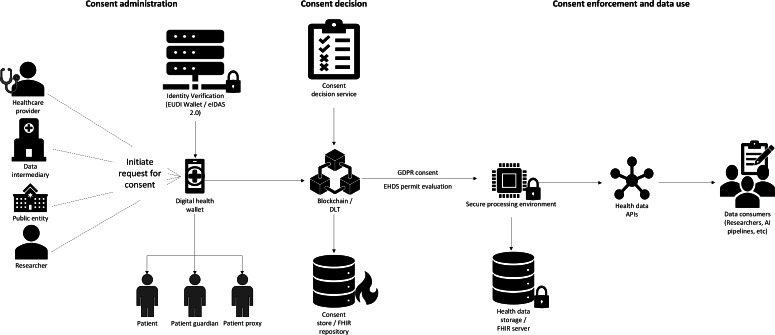
Multilayer framework for implementing dynamic consent in Europe. AI: artificial intelligence; API: application programming interface; DLT: Distributed Ledger Technology; EHDS: European Health Data Space; eIDAS: electronic Identification, Authentication and Trust Services; EUDI: European Digital Identity; FHIR: Fast Healthcare Interoperability Resources; GDPR: General Data Protection Regulation.

The architecture does not assume consent as the universal lawful basis for secondary use, but rather as a context-dependent governance signal that operates alongside EHDS permit-based authorization. The operational architecture supporting dynamic consent consists of 3 sequential stages: consent administration, consent decision, and consent enforcement and data use. Together, they describe how consent is captured, interpreted, and applied within GDPR- and EHDS-compliant environments. These stages align identity verification, interoperable consent artifacts, automated decision logic, and secure processing infrastructures into a unified framework for trustworthy secondary health data use. The overall workflow is illustrated in Figure 1.

Figure 1 provides the conceptual design of the 3-stage architecture, from the capture of consent through to automated decision-making and enforcement mechanisms within SPEs. In this framework, no personal health data are stored directly on-chain; rather, only cryptographic hashes or provenance metadata are recorded, while revocable data remain off-chain under the control of the relevant data holders. This design reduces, but does not fully eliminate, the tension between ledger immutability and data-subject rights such as erasure. In order to operationalize this model, Table 2 gives a detailed breakdown of the actors, components, and processes for each of these stages, expanding out functional elements depicted in that figure and setting out how they together enable dynamic consent across the secondary-use life cycle.

**Table 2.** Functional breakdown of the 3-stage dynamic consent architecture.

Stage	Core actors and components	Key processes	Outputs and compliance contributions
Consent administration	Data subject (patient or guardian or proxy); health care or research initiators; EUDI^a^ Wallet (eIDAS^b^ 2.0); digital health wallet; consent store (FHIR^c^ repository); blockchain	Authentication using high-assurance digital identity; initiation of consent request by authorized actors; granular consent capture through the digital health wallet; encoding consent as an HL7^d^ FHIR^e^ consent artifact; storage of full artifact in consent store; cryptographic hashing and timestamping on DLT for provenance	Authenticated, structured, and machine-readable consent record; tamper-evident evidence of issuance and scope; GDPR^f^ conformity (specificity, informed choice, and revocability)
Consent decision	Consent Decision Service; consent store; blockchain; EHDS^g^ permit evaluation framework	Integrity verification of the consent artifact (signatures, timestamps, and revocation status); cross-checking with DLT for provenance; evaluation of access requests under GDPR; alignment with EHDS permit conditions (SPE^h^ requirement, data minimization, purpose limitation, and export controls); generation of machine-interpretable decision tokens	Authoritative decision token (permit or deny or conditional); harmonized application of individual consent and EHDS governance; traceable decision provenance recorded via DLT references
Consent enforcement and data use	SPEs; health data application programming interfaces; authorized data consumers (research, AI^i^, and public health bodies); auditing and logging infrastructure	Verification and enforcement of decision token at SPE boundary; execution of permitted analytical processes; application of data transformation, minimization, and nonexport safeguards; comprehensive logging of all actions; propagation of consent revocation into enforcement mechanisms	Legally compliant secondary-use operations; complete and tamper-evident audit trail; revocation-aware access control; transparent, accountable data-use environment supporting GDPR and EHDS oversight

a EUDI: European Digital Identity.

b eIDAS 2.0: electronic Identification, Authentication and Trust Services 2.0.

c FHIR: Fast Healthcare Interoperability Resources.

d HL7: Health Level Seven.

e FHIR: Fast Healthcare Interoperability Resources.

f GDPR: General Data Protection Regulation.

g EHDS: European Health Data Space.

h SPE: secure processing environment.

i AI: artificial intelligence.

These 3 stages create, together, an integrated operational pathway where consent is authenticated, encoded in interoperable form, evaluated through transparent legal and technical logic, and enforced within secure environments for secondary health data processing. Through this framework, dynamic consent becomes actionable, verifiable, and revocable throughout its life cycle, while also supporting the rights of the individual and the governance model of the EHDS.

### Integrating the Layers Into a Consent Life Cycle

Dynamic consent must operate as a continuous life cycle rather than a one-time event. Individuals authenticate through trusted digital identities, express preferences through the consent management layer, and have these preferences encoded into standardized artifacts. As data access occurs within SPEs, policy engines enforce the rules. Changes or withdrawals initiated by individuals trigger updates across all relevant systems, and all actions generate audit trails accessible to regulators and, where appropriate, to data subjects. Such updates may be propagated through event-driven notifications, application programming interface–based messaging, and consent-aware policy engines, allowing downstream systems to enforce prospective withdrawal in near real time.

### Implications for European Secondary-Use Governance

The integrated framework aligns dynamic consent with the rights-based ethos of GDPR and with the technical structures introduced by the EHDS. While legal fragmentation and disparate infrastructure remain a challenge, Europe is increasingly set up with interoperable standards, identity tools, and secure processing models that can support dynamic consent. Implementation will be effective only with regulatory and technical coordination across Member States.

The European Commission’s assessment also outlines a series of EU-level actions that would directly support the operationalization of dynamic consent, including the development of a sector-specific Code of Conduct, harmonized interoperability standards, and clearer guidance on pseudonymization and anonymization. Such guidance is particularly important because the legal relevance of dynamic consent depends on whether reused datasets remain personal data under the GDPR or have been transformed into genuinely anonymous information outside its scope. These measures complement the EHDS framework and provide pathways for improving cross-border consistency in consent representation, enforcement, and revocation.

## Discussion

### Principal Findings

The review examined the feasibility of dynamic consent for the secondary use of health data within Europe’s changing regulatory and technical environment. The findings indicate a persistent mismatch between normative expectations introduced by GDPR and practical limitations established by cross-border, data-intensive research settings. As illustrated in the “Critical Analysis: Legal Barriers and Enablers of Dynamic Consent” section, legal fragmentation, plural national understandings of consent requirements, and the institutional governance model brought about by EHDS significantly restrict the function of consent as a general authorization mechanism for secondary use. At the same time, the same analysis also identified key enablers, including the rights-based framing of GDPR and the emerging developments related to interoperability, identity management, and secure data processing.

The implementation framework proposed in the “Implementing Dynamic Consent in Europe” section responds directly to these constraints. By integrating trusted digital identity, machine-readable consent artifacts, policy-based enforcement, SPEs, and transparent auditability, the framework illustrates how dynamic consent can operate within the hybrid governance ecosystem shaped by GDPR and the EHDS. This approach does not replace the institutional authorization workflows but rather positions dynamic consent as a complementary, context-dependent mechanism able to enrich transparency, support user engagement, and strengthen trust in data-driven health systems.

The role of the DGA further reinforces this hybrid perspective. While the EHDS centers institutional authorization through HDABs and SPEs, the DGA supports forms of voluntary, trust-based data sharing through data altruism and intermediary governance. Dynamic consent is especially well suited to this latter domain because it can provide an operational mechanism for communicating preferences, documenting permission, and maintaining participant engagement over time. Integrating this perspective helps situate dynamic consent within a broader EU governance ecosystem rather than treating it solely as a GDPR-derived consent tool.

Empirical insights from the Commission’s study highlight that stakeholders across Europe view current access procedures as complex, costly, and insufficiently harmonized. At the same time, respondents strongly support EU-level harmonization measures to strengthen transparency, facilitate research access, and enhance citizens’ understanding of how their data are reused. These perspectives underscore the importance of aligning dynamic consent models with broader regulatory and infrastructural reforms rather than treating consent as an isolated mechanism [3].

From a policy perspective, these findings highlight the need for greater harmonization across Member States. This need for harmonization is particularly acute during the EHDS transition period, as full operational deployment of HDABs, interoperability frameworks, and SPEs will occur gradually over several years rather than immediately following the regulation’s adoption. It is difficult to scale dynamic consent at an EU level with inconsistent approaches to the interpretation of explicit consent, lawful bases for research, and procedures regarding revocation. The EHDS introduces an element of standardization, particularly through SPEs and harmonized access permits, but guidance from the European Data Protection Board will be important to outline how dynamic consent should work alongside institutional authorization.

The technical analysis underlines the growing potential represented by the emerging digital identity frameworks, such as EUDI Wallet and verifiable credentials, to securely authenticate consent decisions. Similarly, the growing adoption of HL7 FHIR-based consent models and privacy-preserving computation offers promising pathways toward consistent representation and enforcement of dynamic consent across systems. However, the variable maturity of these technologies and real-time policy engine deployment continues to be areas that need to be addressed prior to scaled, reliable implementation of dynamic consent.

Finally, dynamic consent holds important implications for public trust and user experience [20]. As individuals become increasingly aware of how their health data are reused, the ability to modify preferences over time, receive transparent notifications, and access audit logs may strengthen confidence in digital health infrastructures [11]. This aspect is particularly relevant for domains where the sensitivity is high or where the data directly originate from individuals, such as personal health records or citizen-generated data ecosystems. More generally, dynamic consent is best seen as a valuable but context-dependent governance mechanism: one that can enhance transparency and autonomy, provided it is underpinned by harmonized legal interpretation, interoperable technical infrastructures, and robust institutional governance. These are the elements needed to allow its full potential to be realized within Europe’s emerging digital health landscape.

The distinction between pseudonymization and anonymization is also central to the realistic scope of dynamic consent. Dynamic consent remains legally meaningful where secondary-use datasets are still personal data, including where they are pseudonymized and subject to ongoing governance controls. However, where data have been rendered truly anonymous such that reidentification is no longer reasonably possible, GDPR-based rights no longer apply to that dataset, and withdrawal of consent ceases to function as a legal control over subsequent use. Because this threshold is difficult to achieve consistently in many health data contexts, especially where linkage risks persist, dynamic consent remains most relevant in environments where data continue to fall within the law of personal data.

Several existing implementations illustrate how dynamic consent is being applied in practice, although currently limited to specific and context-dependent settings. For instance, digital consent platforms used in biobank governance enable participants to modify their consent preferences over time and receive updates on how their data are being used. Similarly, patient-facing portals in genomic and longitudinal cohort studies allow individuals to manage participation and adjust consent choices dynamically through web-based interfaces.

In addition, initiatives such as the CTRL platform evaluated by Haas et al [21] demonstrate how dynamic consent can be operationalized through interactive digital tools that support ongoing engagement and communication between participants and researchers. While such systems show technical feasibility and improved transparency, their impact on user engagement and decision-making remains mixed, reinforcing the need for further empirical evaluation. These examples highlight that dynamic consent is already being explored in real-world contexts, particularly in research and biobank settings, but its scalability to large-scale, cross-border infrastructures such as those envisaged under the EHDS remains an open question.

Despite its conceptual appeal, dynamic consent remains supported by relatively limited empirical evidence regarding its effectiveness and user acceptance in real-world settings. While prior conceptual and evaluative work has outlined its potential benefits and proposed frameworks for implementation [5,8], recent evaluations suggest that dynamic consent platforms do not necessarily outperform traditional consent models in terms of user engagement or understanding. For example, Haas et al [21] found no significant advantage of a dynamic consent interface over conventional consent approaches in a cardiovascular genetics cohort, highlighting the need for more robust empirical validation.

Furthermore, there is still insufficient evidence on how different populations interact with dynamic consent mechanisms, including their preferences for granular control, frequency of engagement, and long-term usability. Issues such as consent fatigue, cognitive burden, and varying levels of digital literacy may influence adoption and effectiveness, particularly in large-scale public health or cross-border research contexts.

At the same time, several pilot implementations illustrate the emerging practical application of dynamic consent approaches. Examples include digital consent platforms integrated with biobank governance systems, patient portals enabling ongoing preference management, and research-specific interfaces that allow participants to modify consent choices over time. While these initiatives demonstrate technical feasibility, they remain limited in scale and context, and their broader impact on governance and trust is still under evaluation.

Accordingly, dynamic consent should be understood as a promising but still evolving sociotechnical model rather than a fully validated solution. Its future role in EU health data governance will depend not only on legal and technical alignment but also on empirical evidence demonstrating its usability, scalability, and added value compared to existing consent approaches.

To further illustrate the practical implications of dynamic consent, 2 typical use scenarios can be considered. First, in a cross-border research consortium operating under the EHDS framework, a patient provides consent through a digital health wallet for the use of their data in a specific research project. The consent is encoded as a machine-readable artifact (eg, HL7 FHIR Consent), evaluated alongside HDABs’ permits, and enforced within a secure processing environment. If the patient withdraws consent, a revocation signal is propagated through policy engines and access control systems, restricting future use while acknowledging that previously processed data may not be fully retractable.

Second, in a biobank or longitudinal cohort setting, participants interact with a web-based portal that allows them to adjust consent preferences over time, such as opting in or out of specific research domains. These changes are recorded and linked to data access governance processes, enabling more granular and transparent control over participation while supporting ongoing engagement with research initiatives.

These typical cases demonstrate how dynamic consent can function in practice across both institution- and participant-driven contexts, while also highlighting the technical and governance challenges associated with revocation, interoperability, and large-scale implementation.

The implementation of dynamic consent also carries financial, administrative, and social costs. Deploying interoperable identity systems, consent repositories, policy engines, and SPEs requires substantial investment and cross-institutional coordination. In addition, frequent consent requests may contribute to consent fatigue, while digitally mediated interfaces may disadvantage individuals with lower digital literacy or reduced access to digital tools. These constraints should be recognized as practical barriers to equitable deployment.

### Policy and Research Agenda

The findings of this article have several implications for EU-level policy and for future research on health data governance.

First, regulatory guidance on the interaction between consent and institutional authorization is needed. The coexistence of GDPR-based consent, Member-State research exemptions and the EHDS permit model creates uncertainty for controllers and infrastructures seeking to rely on dynamic consent. The European Data Protection Board and the European Commission could help address this by clarifying (1) in which secondary-use scenarios consent remains an appropriate or preferred legal basis; (2) how dynamic consent should be interpreted in light of Articles 6, 9, and 89 GDPR; and (3) how consent-based governance should interface with HDABs’ authorizations and SPEs.

Second, harmonization of consent representation and revocation mechanisms should be prioritized. EU-level work on a common HL7 FHIR Consent profile for secondary use, including standardized codes for purposes, actors, time limits, and withdrawal conditions, would significantly reduce fragmentation. Coupled with guidance on how revocation signals should be propagated across controllers and processors, such standardization would make dynamic consent technically more feasible and legally more predictable.

Third, investment in interoperable digital identity and audit infrastructures is essential. The integration of the EUDI wallet into health-sector workflows and its alignment with consent management platforms would provide the necessary assurance for cross-border authentication of consent decisions. Likewise, the development of robust, possibly tamper-evident, audit mechanisms capable of linking access events to specific consent artifacts would enhance transparency and accountability, whether or not dynamic consent is used.

Fourth, empirical research on user experience, equity, and institutional practice is required. Dynamic consent is not only a legal and technical construct but also a sociotechnical one. Future studies should therefore examine how different populations understand and use dynamic consent interfaces, whether such mechanisms risk exacerbating digital divides or consent fatigue, and how institutions manage the operational burden of implementing granular consent controls. Comparative research across Member States could illuminate how national derogations and institutional cultures affect the uptake and effectiveness of consent-based governance.

Finally, interdisciplinary experimentation with pilot implementations should be encouraged. Controlled pilots that combine EUDI-based identity, FHIR-encoded consent artifacts, policy-as-code enforcement, and EHDS-compatible SPEs would provide valuable evidence on the feasibility, costs, and benefits of dynamic consent in real-world settings. Such pilots could inform future legislative refinements and standardization efforts, ensuring that any broader roll-out of dynamic consent mechanisms is grounded in practical experience rather than purely theoretical expectation.

### Conclusion

Secondary health data use is increasingly at the core of Europe’s ambitions for innovation, evidence-based policy-making, and the development of advanced digital health solutions. Dynamic consent offers a compelling case for strengthening patient autonomy, transparency, and engagement in this shifting landscape. However, this review shows that whether dynamic consent applies in the European context depends on complex legal, technical, and governance issues.

Rather than embedding a fully developed model of dynamic consent, the GDPR provides the legal parameters within which such approaches may be operationalized, particularly through its emphasis on informed, specific, and revocable decision-making; however, this model is constrained in practice by national derogations, diverging interpretations of lawful bases for processing, and a lack of operational clarity in respect of how consent withdrawal should operate across distributed and cross-border data ecosystems. Meanwhile, the EHDS injects a governance model that relies more heavily on institutional authorization, rearranging rather than removing the place of consent in secondary-use workflows. This role is further shaped by the DGA, particularly in data altruism and intermediary-based models where voluntary participation and trust-based reuse remain central. Dynamic consent, in other words, assumes a position of maximum influence in contexts where persons are active contributors or managers of their own data and where transparency and trust are paramount.

Technical gaps remain significant. Limited deployments of interoperable consent standards, uneven rollout of digital identity frameworks, and the absence of robust policy engines capable of real-time enforcement create barriers. However, emerging tools such as FHIR-based consent artifacts, EU digital identity infrastructures, and SPEs provide promising foundations for more consistent and enforceable consent mechanisms.

Consistent with the European Commission’s assessment, this review concludes that significant discrepancies in Member State rules and access pathways impede the scalability of consent-based governance for secondary data use. Coordinated EU-level guidance and harmonized technical standards are therefore essential to support dynamic consent in practice and to ensure its meaningful integration within the hybrid governance model shaped by the EHDS.

Ultimately, the way forward is one that will entail coordinated action. Regulators need to better clarify how consent, research exemptions, and institutional access pathways interlink. Health systems and technology providers must invest in interoperable consent infrastructures and align implementation strategies with evolving identity and audit standards. Governance frameworks also must allow for a balanced approach, integrating individual rights with the operational realities of secondary-use ecosystems.

Dynamic consent cannot itself resolve all the challenges associated with the secondary use of health data, nor is it intended to replace institutional oversight; it should be understood rather as a key component of a multilayered governance ecosystem—one enhancing transparency, improving trust, and promoting meaningful patient participation. With appropriate regulatory clarification, technical standardization, and sustained institutional commitment, dynamic consent has the potential to play a transformative role in Europe’s digital health future.

## Abbreviations

DGA: Data Governance Act
EHDS: European Health Data Space
eIDAS 2.0: electronic Identification, Authentication and Trust Services 2.0
EUDI: European Digital Identity
FHIR: Fast Healthcare Interoperability Resources
GDPR: General Data Protection Regulation
HDAB: Health Data Access Body
HL7: Health Level Seven
PRISMA: Preferred Reporting Items for Systematic Reviews and Meta-Analyses
SPE: secure processing environment
